# Decrypting the Immune Symphony for RNA Vaccines

**DOI:** 10.3390/vaccines13080882

**Published:** 2025-08-20

**Authors:** Brian Weidensee, Itishri Sahu

**Affiliations:** 1 Center for Oncological Research (CORE), Faculty of Medicine and Health Sciences, University of Antwerp, 2610 Antwerpen, Belgium; brian.weidensee@uantwerpen.be; 2 Ziphius NV, Zwijnaarde, 9052 Ghent, Belgium

**Keywords:** RNA vaccines, innate and adaptive immunity

## Abstract

Messenger RNA (mRNA) vaccine technology has revolutionized the field of immunization, offering a non-infectious, non-genome-integrating platform that addresses many limitations of traditional vaccine modalities. Recent advancements in chemical modifications, delivery systems, and manufacturing processes have enhanced the stability, efficacy, and safety of RNA-based therapeutics, expanding their application beyond infectious diseases to include genetic disorders, cancer, and rare diseases. Central to the success of RNA vaccines is their ability to orchestrate a finely tuned immune response, leveraging both innate and adaptive immunity to achieve robust and durable protection. This review synthesizes current knowledge on the immunological mechanisms underpinning RNA vaccine efficacy, with a focus on the roles of pattern recognition receptors (PRRs) such as Toll-like receptors (TLRs) and RIG-I-like receptors (RLRs) in sensing exogenous RNA, the impact of RNA modifications and manufacturing impurities on innate immune activation, and the subsequent cytokine and chemokine milieu that shapes adaptive responses. We also discuss the dual role of lipid nanoparticle (LNP) delivery systems as both carriers and adjuvants, highlighting their contribution to the vaccine’s immunogenicity and reactogenicity profile. Understanding these complex immune interactions is critical for optimizing RNA vaccine design, minimizing adverse effects, and expanding their therapeutic potential. This review aims to provide a comprehensive overview of the immune symphony orchestrated by RNA vaccines and to identify key areas for future research to further refine and expand the utility of this transformative technology.

## 1. Introduction

mRNA-based technology has emerged as a breakthrough platform for developing vaccines in comparison to killed or live attenuated viruses, DNA-based vaccines, and protein subunit vaccines. The non-infectious, non-genome-integrating technology provides safety from the potential risk of infections or insertional mutagenesis [1]. Simultaneously, the advancement with regard to chemical modifications, formulations, and delivery has provided the flexibility needed to control inherent immunogenicity and reactogenicity and regulate the half-life of active agents [1]. These have provided the community an opportunity to further explore them in therapeutic applications such as protein replacement therapy and gene editing [2].

The emergency use authorization (EUA) of mRNA-based vaccines against COVID-19 was quickly followed up by a tremendous increase in preclinical data by numerous labs and pharmaceutical companies to leverage it further for other disease conditions. A quick search in the ClinicalTrials.gov database indicates around 400 RNA-based clinical trials that are in various stages, indicating the paradigm shift [2]. However, for the purpose of this paper, we would like to explore the intricacies involved in the immune orchestra, which plays a major role in making this new technology a success, and also the areas which need further decryption to learn and modulate the technology to make it even more suitable for different indications.

Advancements in RNA technology have significantly enhanced its stability and efficacy while simultaneously expanding its potential for therapeutic applications beyond vaccines [3,4]. These developments have been driven by innovative approaches in chemical modification, delivery systems, and manufacturing processes, which have collectively addressed many of the initial challenges associated with RNA-based therapies [3,4,5]. The improved stability of RNA molecules has extended their half-life in biological systems, allowing for sustained therapeutic effects and potentially reducing the frequency of administration required for treatment [3,4,5]. As listed in Table 1, these technological breakthroughs have facilitated the emergence of RNA-based treatments in diverse areas, including genetic disorders, cancers, and rare diseases, thereby demonstrating the versatility and potential of this platform [5,6,7]. In genetic disorders, RNA therapeutics offer the possibility of addressing previously untreatable conditions by modulating gene expression or correcting genetic defects at the RNA level [4,5,7]. RNA-based approaches are being explored for targeted gene silencing, immunomodulation, and personalized vaccine development for cancer treatment [4,5,6,7]. In the field of rare diseases, where traditional drug development often faces economic challenges, RNA therapeutics presents a promising avenue for developing tailored treatments for small patient populations [5,7].

## 2. Rationale for Evaluating Immune Responses

Despite transformative progress, RNA-based therapeutics and vaccines have demonstrated that their interaction with the immune system can lead to safety concerns, including significant innate immune activation, strong inflammatory responses, and, in rare cases, serious adverse effects such as myocarditis, anaphylaxis, or neurological complications. Local and systemic reactogenicity, immunogenicity that may limit repeated dosing, and unpredictable individual tolerability remain key issues in both clinical trials and real world deployment [14,15,16].

Given these challenges, it is becoming increasingly imperative to understand the complex interplay between these molecules and the immune system [3,4,9]. This understanding is crucial for several reasons. First, the immune response to RNA can significantly affect the safety and efficacy of these therapies. Second, harnessing the immunomodulatory properties of RNA could potentially enhance the therapeutic outcomes. Finally, mitigating unwanted immune reactions could improve the tolerability and long-term use of RNA-based treatments. Elucidating the intricate mechanisms of innate immune responses to RNA-based interventions is crucial for optimizing their safety profiles and enhancing their therapeutic efficacy across a broad spectrum of medical applications. This involves investigating the various pattern recognition receptors involved in RNA sensing, signaling cascades triggered by RNA recognition, and subsequent cellular and humoral immune responses.

Furthermore, understanding how different RNA modifications and delivery systems influence immune interactions is essential for designing more effective and safe RNA therapeutics. Ongoing research in this field is likely to uncover new insights into the fundamental biology of RNA–immune system interactions, potentially leading to novel therapeutic strategies that leverage these mechanisms [3,4,9]. As our knowledge deepens, it may be possible to fine-tune RNA-based therapies to achieve the desired immunomodulatory effects while minimizing adverse reactions, thereby expanding the therapeutic window and applicability of these innovative treatments across various medical disciplines.

## 3. RNA Vaccines and Fundamental Immunological Principles

Vaccination fundamentally relies on the coordinated activation of both the innate and adaptive branches of the immune system to elicit protective responses. The immune system comprises two interconnected branches: innate immunity, which provides immediate, non-specific defense, and adaptive immunity, characterized by its antigen specificity and the capacity to generate long-lasting immunological memory [17]. An effective vaccine must engage both arms of this system to initiate and sustain protective responses.

Upon administration, vaccine components are detected by innate immune cells, triggering a cascade of signaling events. These cells not only help contain the perceived threat but also process and present antigens to the adaptive immune system. This primary response establishes a foundation of memory cells and antibodies, ensuring a faster, more robust, and highly specific response upon subsequent exposure to the actual pathogen, thereby preventing or mitigating disease severity [18,19].

RNA vaccines operate through a streamlined yet highly effective mechanism. Once administered, the mRNA, typically encapsulated within a protective delivery vehicle such as Lipid Nanoparticles (LNPs), is taken up by host cells, predominantly antigen-presenting cells (APCs) like dendritic cells [20,21]. Inside the cytoplasm, the mRNA serves as a blueprint for the cell’s ribosomes to translate it into the target antigen protein. This in situ production of antigens is crucial, as it mimics aspects of natural infection without the risks associated with live pathogens. The newly synthesized antigen is then processed and presented on the cell surface via Major Histocompatibility Complex (MHC) molecules, activating naive T and B lymphocytes and thereby initiating the adaptive immune response that culminates in protective immunity [22].

## 4. Innate Immune Responses: Piano

The initiation of a robust adaptive immune response is tightly regulated by the early activation of the innate immune system. The recognition of foreign RNA by innate sensors plays a pivotal role in shaping the magnitude and quality of the ensuing adaptive response [23]. This initial phase, often characterized by a controlled inflammatory response, provides the necessary signals and cellular context for the subsequent development of specific and durable immunity. RNA vaccines harness this natural recognition system to enhance immunogenicity while requiring careful modulation to avoid excessive inflammatory responses [20]. Striking the right balance between immune activation and tolerance is therefore a critical aspect in RNA vaccine design [24].

### 4.1. RNA Sensing and Pattern Recognition Receptors (PRRs)

The host immune system possesses an intricate network of Pattern Recognition Receptors (PRRs) evolved to detect conserved molecular patterns linked to pathogens (pathogen-associated molecular patterns, PAMPs), including various forms of RNA. In the context of RNA vaccines, both the synthetic mRNA payload and components of the delivery system can engage these PRRs, thereby triggering innate immune signaling. Although many PRRs are present within the innate immune system, this paper will focus only on relevant molecules involved in recognizing RNA vaccines.

**Figure 1:**
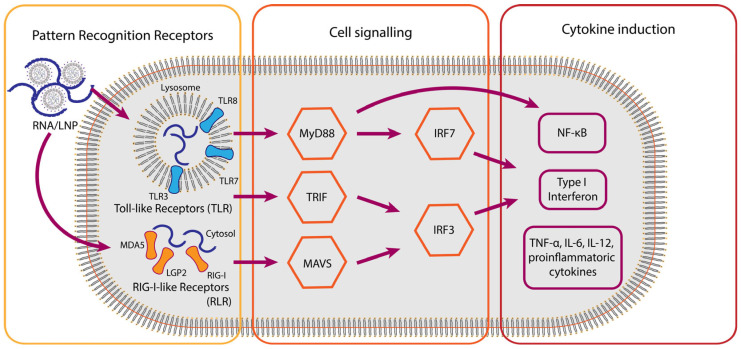
Schematic representation of RNA sensing by the innate immune system; the RNA that entered the cell is recognized by Pattern Recognition Receptors (PRRs), followed by downstream cell signaling and cytokine induction leading to an inflammatory response and immune activation.

Toll-like Receptors (TLRs) are a crucial family of PRRs, with specific members recognizing different types of nucleic acid structures. TLR3 is mainly located in endosomes and detects double-stranded RNA (dsRNA), a common intermediate in viral replication [25]. In contrast, TLR7 and TLR8, also found within endosomal compartments, specialize in recognizing single-stranded RNA (ssRNA), especially uridine-rich or GU-rich sequences (Figure 1) [17]. The activation of these TLRs by vaccine-derived RNA is a key step in triggering the innate immune response, leading to a cascade of downstream signaling events that produce type I interferons and other pro-inflammatory cytokines [17]. Interestingly, studies have shown that COVID-19 mRNA vaccine (BNT162b2)-induced antibody and T cell responses do not rely on signaling through TLRs 2, 3, 4, 5, and 7, suggesting that other pathways are more important [26]. Furthermore, mRNA vaccines are designed to avoid TLR activation by using capping and nucleoside modifications, highlighting the importance of controlling the immunogenicity of RNA vaccines.

Beyond the endosomal TLRs, the cytoplasm of host cells is equipped with another critical family of RNA sensors, the RIG-I-like Receptors (RLRs), which include RIG-I, MDA5, and LGP2 [25]. These receptors function as cytoplasmic sensors of (viral) RNA, initiating and modulating immunity. RIG-I is specifically activated by short dsRNAs featuring 5′-diphosphate or 5′-triphosphate ends and lacking 2′-O-methylation, which are characteristic of non-self RNA [27,28]. The employment of the 7-methyl guanosine cap structure in combination with 2′-O-methyl modifications facilitates the recognition as self RNA. It can therefore be employed as a method to protect RNA vaccines from recognition by RIG-I, followed by innate immune activation [29,30]. MDA5, on the other hand, prefers to sense longer dsRNAs, typically exceeding 500 base pairs, and does not require specific triphosphate ends for its activation [25]. MDA5 activates downstream signaling through the mitochondrial antiviral-signaling protein (MAVS), leading to the activation of transcription factors such as IRF3 and NF-κB (Figure 1) [26,31].

### 4.2. The Role of RNA Modification and Manufacturing in Innate Immune Activity

A critical design element in the development of highly effective mRNA vaccines, such as those used for COVID-19, is the incorporation of nucleoside modifications, most notably pseudouridine (Ψ) and its derivative N1-methylpseudouridine (m1Ψ). These modifications significantly enhance the translational efficiency and stability of the mRNA by reducing recognition by innate immune pattern recognition receptors (PRRs), such as Toll-like receptors (TLR7/8) and cytosolic receptors like RIG-I and MDA5, allowing for greater antigen production within host cells [23]. This reduction in innate immune sensing prevents the activation of downstream inflammatory pathways that could otherwise lead to rapid mRNA degradation via RNase L activation or translation inhibition through protein kinase R (PKR) signaling, thereby facilitating greater and more sustained antigen production within host cells. Karikó et al. (2008) first demonstrated that pseudouridine incorporation into synthetic RNA reduces the activation of endosomal TLRs, enabling a stealth-like effect that enhances protein expression and vaccine tolerability [32]. Subsequent studies confirmed that m1Ψ-modified mRNAs outperform pseudouridine-containing counterparts with improved translational efficiency and reduced innate immune activation, thereby improving the vaccine safety profile and immunogenic potency.

This strategic dampening of PRR activation is a deliberate design choice aimed at curbing an excessive or unwanted inflammatory response that could otherwise impair antigen expression and cause adverse reactogenicity. Notably, despite these modifications, mRNA-LNP vaccines continue to elicit robust innate immune activation, reflecting the complex interplay between the mRNA payload and the immunostimulatory properties of the lipid nanoparticle carrier [26,33,34]. Importantly, this engineered immunogenic “Goldilocks zone”—where the mRNA is stable and sufficiently “invisible” to avoid premature recognition but not entirely immunologically silent—is critical for eliciting balanced innate immune signaling that effectively primes adaptive immunity without deleterious inflammation [35].

However, the modulation of innate immune activation by RNA modifications alone is insufficient to fully control the immunogenicity of RNA vaccines; during in vitro transcription (IVT), the enzymatic reaction that synthesizes mRNA, double-stranded RNAs (dsRNAs) can inadvertently form as byproducts through template switching, self-complementarity, or abortive transcription events. These dsRNA contaminants are potent ligands for innate immune receptors, particularly melanoma differentiation-associated protein 5 (MDA5) and TLR3, triggering strong type I interferon and pro-inflammatory cytokine responses [36]. Their presence can exacerbate innate immune activation beyond the desired threshold, potentially increasing systemic reactogenicity and influencing the nature of ensuing adaptive responses. Nelson et al. (2020) quantitatively demonstrated a correlation between dsRNA impurity levels and heightened interferon signaling in vitro and in vivo [37].

Consequently, advanced purification methods, such as high-performance liquid chromatography (HPLC) and cellulose chromatography, have been developed and increasingly implemented to selectively remove these dsRNA contaminants from mRNA preparations, thereby enhancing safety and yielding more consistent innate immune activation profiles [38].

Therefore, the interplay of deliberate RNA chemical modifications and stringent manufacturing purity is essential to optimize the immune profile of RNA vaccines. Achieving a balance wherein the mRNA construct and its delivery system provoke sufficient innate immune stimulation to initiate effective antigen presentation and adaptive immune priming, while minimizing excessive inflammation that could degrade mRNA or produce adverse effects, remains a critical and active area of vaccine design. This dual modulation of innate immunity through RNA structure and manufacturing quality underscores the sophisticated engineering behind modern RNA vaccine platforms.

### 4.3. Interferon Production and Early Signalling Pathways

The activation of PRRs by RNA vaccine components rapidly culminates in the production of interferons, particularly Type I Interferons (IFN-α, IFN-β), which are central to the early immune response and the subsequent shaping of adaptive immunity (Figure 1).

Upon the recognition of RNA ligands, TLRs (via MyD88 or TRIF pathways) and RLRs (via MAVS) trigger complex intracellular signaling cascades that lead to the activation of transcription factors such as IRF3 and IRF7, as well as NF-κB [31]. These transcription factors translocate to the nucleus, where they induce the expression of genes encoding Type I interferons (IFN-α and IFN-β). Type I IFNs are pleiotropic cytokines that establish an immune-active state in infected and neighboring cells (particularly as a response to viral infection), upregulate MHC molecule expression, and promote the maturation and activation of professional APCs like dendritic cells, thereby bridging the innate and adaptive immune responses [39].

In comparison, IFN-γ is classically associated with adaptive T cell responses, particularly from Th1 cells and cytotoxic T lymphocytes (CTLs); it also plays a role in early innate immune activation following vaccination. Natural Killer (NK) cells are significant contributors to early IFN-γ production after the first vaccine dose [40]. This early IFN-γ signalling can activate antigen-presenting cells and contribute to the inflammatory milieu that primes adaptive immunity [40].

### 4.4. Cytokine and Chemokine Profiles

The innate immune activation induced by RNA vaccines generates a distinct cytokine and chemokine profile, which acts as a molecular “symphony” guiding the subsequent adaptive immune response. mRNA-LNP vaccines are potent inducers of a range of pro-inflammatory cytokines and chemokines. These include Interleukin-1 beta (IL-1β), Interleukin-6 (IL-6), Tumour Necrosis Factor-alpha (TNF-α), Leukaemia Inhibitory Factor (LIF), Granulocyte-Macrophage Colony-Stimulating Factor (GM-CSF), Interferon-alpha (IFN-α), and Interferon-gamma (IFN-γ) [41,42]. Additionally, various CC- and CXC-motif chemokines such as CXCL1, CXCL2, CXCL5, CXCL10, CCL3, and CCL4 are rapidly stimulated [40,42]. This robust inflammatory signature is largely driven by the ionizable lipid component of the LNPs, which possesses intrinsic adjuvant properties [40]. The ionizable lipid component of LNPs is a critical determinant of the vaccine’s ability to induce an immune response. Highly purified mRNA, even with nucleoside modifications, when combined with LNPs lacking this inflammatory lipid, is unable to induce robust innate and adaptive immune responses in vivo in mice [33,43]. This highlights that while mRNA carries the antigenic blueprint, the LNP acts as the primary orchestrator of the innate immune response, generating the necessary inflammatory environment for effective adaptive immune priming. The acute reactogenicity responses commonly observed after vaccination, such as fever, headache, fatigue, myalgia, and chills, are direct manifestations of this rapid and high-level release of innate inflammatory cytokines upon exposure to the LNPs [44]. This demonstrates that the reactogenicity is not merely an undesirable side effect but an indicator of the deliberate, LNP-driven innate immune activation that is fundamental to the vaccine’s immunogenicity.

The innate immune response to mRNA vaccination can be significantly modulated by an individual’s prior immunological history. Studies have shown that prior SARS-CoV-2 infection confers a significantly stronger induction of proinflammatory and type I IFN-related gene signatures, as well as higher levels of serum cytokines and increased monocyte expansion, following the prime (first) mRNA vaccination [45]. This augmentation of the innate response is correlated with pre-existing antigen-specific CD4 T cells, antibodies, and memory B cells, indicating that adaptive memory formed by infection can enhance vaccine-induced innate immune activation [45]. Furthermore, sequential vaccination also influences the innate response. The second vaccination typically leads to a further increase in the magnitude of the early innate response in both individuals with and without prior infection. However, a third vaccination does not appear to further increase vaccine-induced inflammation [45].

This suggests a complex interplay where adaptive memory, whether from natural infection or previous vaccination, can “prime” the innate immune system, leading to a more robust initial response upon subsequent antigen exposure. This phenomenon, often referred to as “hybrid immunity” when combining natural infection and vaccination, can result in a qualitatively different innate response, potentially contributing to enhanced protection or altered reactogenicity profiles compared to vaccination in naive individuals.

## 5. Innate Cellular Immunity

Beyond soluble mediators, innate immune cells play direct and crucial roles in shaping the response to RNA vaccines, from antigen processing to direct effector functions.

### 5.1. Dendritic Cells (DCs)

Dendritic cells (DCs) are widely recognized as the most potent antigen-presenting cells (APCs) and are central to initiating adaptive immunity [46]. In the context of mRNA vaccination, DCs efficiently take up the mRNA-LNP complexes, primarily through phagocytosis. Once internalized, the mRNA is released into the cytoplasm and translated into the target antigen protein. This newly synthesized protein is then processed by the cell’s proteasomes, and the resulting peptides are loaded onto MHC Class I and Class II molecules [46]. These MHC–peptide complexes are then transported to the DC surface, effectively “activating” the DC. Activated DCs subsequently migrate to draining lymph nodes, where they present the antigen to naive T cells and B cells, providing the critical signals necessary to initiate and shape the adaptive immune response [47]. The efficiency of mRNA uptake and antigen presentation by DCs is a key determinant of vaccine immunogenicity.

### 5.2. Macrophages and Monocytes

Macrophages and monocytes, crucial components of the innate immune system, are also significantly activated by mRNA vaccines. These cells undergo profound epigenetic and transcriptomic changes following vaccination, leading to a sustained pro-inflammatory immune response [48]. This long-term adaptation of innate immune cells, known as “trained immunity” or “innate immune memory,” enables them to respond more effectively and robustly to subsequent antigen exposures, even those unrelated to the original stimulus [48].

The induction of trained immunity by mRNA vaccines signifies a deeper and more persistent impact on the innate immune system than previously appreciated. This involves epigenetic modifications and the metabolic reprogramming of innate cells, allowing them to mount an enhanced and rapid response to various pathogen-associated molecular patterns (PAMPs) [48]. This implies that the benefits of mRNA vaccination may extend beyond antigen-specific adaptive memory, potentially conferring broader, non-specific protection against a range of unrelated pathogens or influencing responses to subsequent inflammatory challenges. This emerging understanding opens new avenues for vaccine design, aiming to leverage innate memory for enhanced and broader protective immunity.

### 5.3. Natural Killer (NK) Cells

Natural Killer (NK) cells are innate lymphoid cells that play a vital role in the early defense against viral infections and cancer. In the context of mRNA vaccination, NK cells contribute to the early production of IFN-γ, a cytokine crucial for antiviral immunity and immune cell activation [46]. Beyond their direct effector functions, there is growing interest in leveraging NK cells for therapeutic applications. Research is actively exploring methods for efficient in vivo mRNA delivery, specifically to splenic NK cell subsets, aiming to develop novel NK cell-based immunotherapies for cancer and viral diseases [49]. This highlights the potential of mRNA technology to not only induce adaptive immunity but also directly modulate and enhance the functions of key innate effector cells.

## 6. Adaptive Immune Response: Crescendo

An adaptive immune response functions by destroying invading pathogens and or toxic molecules produced by them. These can be classified into two major categories: antibody-based and cell-mediated, which are carried out by different classes of Lymphocytes B cells and T cells. For any vaccine, it is the ultimate goal to ensure specificity and immunity, and RNA vaccines excel at orchestrating this adaptive symphony. The adaptive immune recognition relies on the generation of randomly and highly diverse antigen receptors by the recombination of variable (V), diversity (D), and joining (j) segments during the T and B cell receptors rearrangement phase, followed by the clonal selection and amplification of receptors with relevant specificity [50]. Upon a pathogenic encounter, the lymphocytes that can recognize antigens present expand, and some of these differentiate into long-lived memory T and B cells, which then can be reactivated upon subsequent encounters/infections.

### 6.1. Humoral Immunity—B Cell Activation and Antibody Production

Humoral immunity, mediated by B lymphocytes and the antibodies they produce, represents a fundamental arm of vaccine-induced protection, particularly through the neutralization of pathogens and the prevention of infection. The induction of potent humoral responses by mRNA-lipid nanoparticle (LNP) vaccines involves a highly coordinated sequence of B cell activation, proliferation, differentiation, and memory formation [20,51,52].

Initial B cell activation is initiated through the direct recognition of vaccine-encoded antigens by the B cell receptor (BCR), providing the primary signal for activation [20]. This is followed by essential co-stimulation from T follicular helper (Tfh) cells, which play a central role in guiding the development of high-affinity, class-switched antibody responses [53,54]. Tfh cells localize to germinal centers (GCs) within secondary lymphoid organs, where they provide cytokines (e.g., IL-21, IL-6) and co-stimulatory signals that drive B cell proliferation, somatic hypermutation, and affinity maturation [54,55]. The intrinsic adjuvant properties of LNPs, particularly their ionizable lipid components, further promote Tfh differentiation by inducing robust IL-6 responses [56].

mRNA vaccines have been shown to elicit broad polyclonal B cell responses, targeting multiple epitopes of viral antigens, which enhances the breadth and durability of neutralizing antibody responses [57]. This capacity for epitope diversity is especially important for maintaining protection against emerging viral variants [58]. Concurrently, class-switch recombination skews the antibody response predominantly toward the IgG isotype, with limited mucosal IgA production—a pattern reflective of the systemic route of vaccine delivery and its implications for protection against mucosal infections [59,60]. Within GCs, ongoing affinity maturation leads to the production of antibodies with progressively higher binding affinity. This maturation is critical for the development of long-lived plasma cells and high-avidity antibody responses that correlate with improved viral neutralization [61]. Long-lived plasma cells, residing primarily in bone marrow niches, sustain systemic antibody levels for months after vaccination, contributing to durable humoral immunity [20,61]. Factors influencing their development include both the quality of Tfh cell help and the cytokine milieu during early B cell activation [54,62].

Equally important is the generation and persistence of memory B cells, which serve as a reservoir for rapid and enhanced secondary responses upon antigen re-exposure. mRNA vaccines induce robust, class-switched memory B cell populations that continue to evolve over time [52,57]. Notably, booster immunizations can reactivate these memory cells, drive additional rounds of affinity maturation, and further improve antibody quality [53,63]. Studies indicate that memory B cell numbers not only persist but can increase over several months post-vaccination, even as circulating antibody titers naturally decline [64]. These cells are poised to rapidly differentiate into antibody-secreting plasma cells upon their re-encounter with the antigen, ensuring swift recall responses and ongoing protection against severe disease [55]. However, individual factors such as age may influence the quality and diversity of memory B cell responses. In older adults, although initial neutralizing antibody titers may approximate those of younger individuals, their memory B cell compartments tend to be less diverse and more clonally restricted, potentially compromising long-term protective immunity [65].

Together, these findings underscore the intricate interplay between early B cell activation, germinal center dynamics, and the sustained function of memory B cells and plasma cells. Optimizing these processes through rational vaccine design and tailored immunization strategies holds promise for enhancing the durability and breadth of humoral immunity induced by mRNA vaccines.

### 6.2. Cellular Immunity: T Cell Activation

The profound success of mRNA vaccines against COVID underscored their capacity to elicit potent and broad immune responses. A critical component of this protective immunity is the robust activation of cellular immunity. Cellular immunity is led by T lymphocytes, which serve as a foundational layer of defense, particularly vital for combating intracellular pathogens and malignancies. T cells not only eradicate infected or aberrant cells but also regulate and harmonize broader immune mechanisms essential for vaccine-mediated protection.

#### 6.2.1. CD4^+^ T Cell Differentiation and Functional Specialization

Following mRNA vaccination, antigen-presenting cells (APCs) process and display vaccine-encoded peptides via MHC class II, activating naïve CD4^+^ T cells and initiating their differentiation into specialized effector subsets. These include T helper 1 (Th1), Th2, Th17, regulatory T cells (Tregs), and T follicular helper (T_FH) cells, each contributing uniquely to the immune architecture.

Notably, mRNA vaccines favor a Th1-biased polarization, promoting the secretion of IFN-γ, IL-2, and TNF-β, cytokines critical for antiviral defense and tumor surveillance [66,67,68]. This Th1 skew is considered beneficial, as it minimizes the risk of Th2-driven hypersensitivity or allergic responses, which are primarily mediated by IL-4, IL-5, and IL-13 [69]. Though less prominent, Th17 cells, shaped by TGF-β and IL-6, secrete IL-17 and IL-22 and contribute to mucosal immunity and inflammatory modulation [70,71]. Meanwhile, Tregs (FoxP3^+^) serve as immunological brakes, preserving tolerance and preventing overactivation. A critical subset of CD4^+^ T cells, the T follicular helper (T_FH) cells, are robustly induced by mRNA vaccines and are instrumental in mediating germinal center formation, B cell affinity maturation, and antibody isotype switching [72]. T_FH abundance post-vaccination correlates closely with the magnitude and quality of neutralizing antibody responses, reinforcing the functional synergy between cellular and humoral immunity.

#### 6.2.2. CD8^+^ T Cell Cytotoxicity and Polyfunctionality

mRNA vaccines also induce potent CD8^+^ cytotoxic T lymphocyte (CTL) responses by enabling antigen cross-presentation via MHC class I on dendritic cells [73]. Upon activation, CTLs utilize a repertoire of effector mechanisms to eliminate virus-infected or transformed cells. These include the perforin–granzyme pathway, which induces apoptosis through pore formation and proteolytic cleavage, as well as Fas–FasL interactions that trigger death receptor-mediated apoptosis. Additionally, CTLs secrete proinflammatory cytokines such as IFN-γ and TNF-α, which possess both direct antiviral effects and immunoregulatory functions [74].

A hallmark of mRNA vaccine-induced CTLs is their polyfunctionality—the capacity to simultaneously produce multiple effector molecules and cytokines, which has been strongly associated with superior protective efficacy [75]. These responses arise rapidly post-vaccination and are detectable even before the peak of antibody titers, suggesting an early window of cellular immunity that contributes to initial viral containment.

#### 6.2.3. Memory T Cell Formation and Clonal Replenishment

Beyond effector responses, mRNA vaccines elicit long-lived memory T cell subsets, including central memory (T_CM), effector memory (T_EM), and tissue-resident memory (T_RM) populations. These memory cells play distinct yet complementary roles in sustaining immune readiness. While T_CM and T_EM recirculate and rapidly mobilize upon re-exposure, T_RM cells, particularly those localized in mucosal and peripheral tissues, provide frontline protection at portals of viral entry [76]. Emerging evidence highlights that systemic mRNA vaccination can induce T_RM cells in non-lymphoid sites such as the nasal mucosa, where CD8^+^ CD69^+^CD103^+^ and CD4^+^ CCR6^+^CD161^+^ T cells accumulate and exhibit cytotoxic functionality [76]. The development and persistence of these cells suggest potential mucosal protection, which is an important consideration for respiratory pathogens like SARS-CoV-2. T cell memory is also dynamically reshaped by booster vaccination, a process described as clonal replenishment, where new clones are recruited, and pre-existing memory populations are reactivated to broaden immune repertoire and durability [77,78,79]. This phenomenon underscores the adaptive flexibility of the T cell response to sequential mRNA vaccine exposures [79,80].

## 7. Memory Induction and Durability: Sustaining the Immune Symphony

In the immune symphony orchestrated by mRNA vaccines, immunological memory acts as the lasting resonance, preserving the melody of protection long after the initial immunogenic exposure. The establishment and maintenance of long-term memory are crucial for durable defense against reinfection, and mRNA vaccines demonstrate a remarkable capacity to induce robust, multifaceted memory responses.

### 7.1. Harmonizing Humoral and Cellular Memory

mRNA vaccines stimulate a coordinated generation of memory B and T cells, forming the foundation of long-term immunity [52]. These memory cells persist long after antigen clearance and can rapidly reactivate upon re-encounter, facilitating swift and effective pathogen elimination. Memory B cells differentiate into long-lived plasma cells, particularly within bone marrow niches, maintaining sustained antibody production that contributes to baseline serological protection [81,82].

On the cellular side, vaccines elicit multiple memory T cell subsets that span the immune landscape. Central memory T cells (T_CM), housed in lymphoid tissues, express CCR7 and CD62L and possess high proliferative potential. Effector memory T cells (T_EM) circulate in peripheral tissues and can execute immediate cytotoxic or cytokine-mediated responses. Importantly, tissue-resident memory T cells (T_RM) remain anchored in non-lymphoid tissues such as the lungs, skin, and gastrointestinal tract, poised for local immune defense at sites of pathogen entry [75]. These T_RM cells play an increasingly recognized role in mucosal immunity and the rapid containment of respiratory viruses such as SARS-CoV-2.

### 7.2. Priming Conditions and Memory Longevity

The quality and longevity of memory responses are intimately linked to the conditions under which immune priming occurs. mRNA vaccines provide a unique advantage by simultaneously activating innate sensors (e.g., Toll-like receptors), facilitating efficient antigen presentation, and promoting germinal center reactions—all of which enhance memory formation [20,43]. Additionally, the cytokine milieu during the early immune response, including IFN-γ and IL-6, shapes the differentiation and stability of both B and T cell memory populations [83]. Although the full duration of mRNA vaccine-induced memory continues to be assessed, current studies demonstrate persistent antibody titers and detectable memory T cell frequencies months after vaccination. These observations suggest that the immune memory induced by mRNA platforms is not only potent but enduring, offering long-term surveillance and rapid recall upon re-exposure [75].

### 7.3. Memory Recall and the Role of Boosting

Booster vaccinations act as conductor’s cues, reactivating dormant immune players and enhancing the overall amplitude and complexity of the immune response. mRNA vaccine boosters are highly effective at recalling memory B cells and expanding memory T cell clones, leading to increased antibody levels and strengthened cellular immunity that often surpasses the original response [52]. Notably, booster doses improve cross-variant protection, crucial for addressing viral evolution. Mechanistically, clonal replenishment occurs with successive doses, where new immune clones are recruited while previously primed populations are re-energized [52,84]. This dynamic adaptability allows the immune system to evolve in response to shifting antigenic landscapes, making mRNA platforms particularly well suited for variant-specific and multivalent vaccine strategies.

### 7.4. Hybrid Immunity

Individuals with prior SARS-CoV-2 infection who subsequently receive mRNA vaccines exhibit hybrid immunity, a phenomenon marked by the heightened magnitude and breadth of memory responses [85,86]. These individuals mount superior CD4^+^ and CD8^+^ recall responses, higher affinity antibody production, and broader variant coverage compared to those with vaccination alone. Hybrid immunity represents a compelling model for how natural and vaccine-induced memory can harmonize, creating a crescendo of protection in the immune orchestra.

## 8. Vaccine Formulation, Delivery, and Safety

### 8.1. Vaccine Formulation and Delivery Systems

The efficacy of RNA vaccines is fundamentally linked to advances in formulation and delivery, particularly through the use of lipid nanoparticles (LNPs), playing a pivotal and multifaceted role. LNPs serve multiple roles: they protect the fragile mRNA from degradation by extracellular ribonucleases and thereby enhance their stability and circulation time in biological environments. Upon administration, LNPs facilitate efficient cellular uptake through endocytosis. Within the acidic endosomal compartments, the ionizable lipids become protonated, including electrostatic interactions that destabilize and disrupt the endosomal membrane, enabling the efficient cytoplasmic release of the RNA payload. Moreover, LNPs create immune sensors such as Toll-like receptors and inflammasomes, acting as adjuvants to potentiate robust innate and adaptive immune responses essential for effective immunogenicity [20,87].

**Figure 2:**
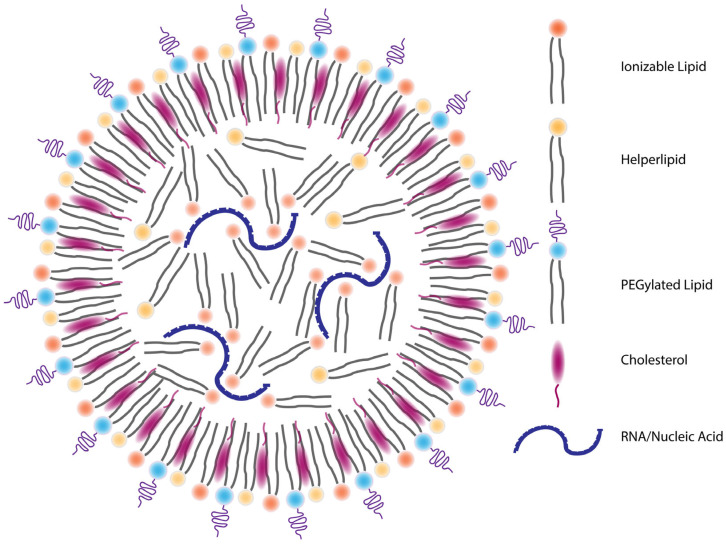
Composition of RNA-LNP for vaccine application. A lipid monolayer is formed by an ionizable lipid that also interacts with the RNA in the center of the LNP; additionally, a helper lipid, a PEGylated lipid, and cholesterol facilitate the formation and stability of the LNP.

The efficacy and safety profile of mRNA vaccines are profoundly influenced by the precise composition and structural design of their LNP delivery systems. Canonical LNPs consist of four main lipid components: ionizable lipids, cholesterol, helper phospholipids, and PEGylated lipids, each contributing unique properties to enhance delivery and immunogenicity (Figure 2) [20]. Ionizable lipids are the cornerstone of LNPs. At acidic pH during formulation, they acquire a positive charge that enables them to complex with negatively charged mRNA. Upon endosomal acidification post-cellular uptake, they again become protonated, promoting endosomal escape via mechanisms such as membrane fusion and osmotic swelling [88]. Their chemical structure, including a tertiary amine headgroup and hydrophobic tails, is designed for high encapsulation efficiency (>90%) and cytosolic release. Ionizable lipids also exhibit adjuvant properties, stimulating innate immune responses through pathways such as TLR4 activation [89]. Cholesterol modulates bilayer fluidity and provides structural integrity, facilitating membrane fusion and LNP stability [20]. Helper phospholipids, such as DSPC, assist in forming the lipid bilayer and enhance nanoparticle rigidity [90]. PEGylated lipids confer colloidal stability and extend circulation time by preventing aggregation and opsonization. However, anti-PEG antibodies may reduce efficacy upon repeated dosing, prompting the development of alternative stealth coatings like poly(carboxybetaine) [91,92,93].

The design of LNPs embodies a “Goldilocks” principle, requiring a delicate balance: the LNP must be stable enough to protect the mRNA cargo from degradation yet labile enough to release it efficiently inside target cells [94,95]. Simultaneously, it needs to be sufficiently shielded to evade immediate immune surveillance but not so hidden that it hinders cellular uptake and subsequent immune activation [95]. This intricate balance extends to the specific chemical properties of the ionizable lipids. For instance, novel zwitterionic materials like poly(carboxybetaine) (PCB) have been developed to replace PEG, offering a superior balance of stealth and stability—reducing protein adsorption and immune activation while still enabling antigen-specific immune responses [35,96]. This continuous refinement of LNP composition and physicochemical properties (such as size, shape, and surface charge) is paramount, as even slight modifications can profoundly alter the LNP’s interaction with biological fluids and immune cells, directly impacting both efficacy and safety [35].

### 8.2. Mechanisms of Uptake, Immune Activation, and Delivery Optimization in RNA Vaccines

#### 8.2.1. Cellular Uptake and Endosomal Escape

For mRNA vaccines to exert their biological effect, the lipid nanoparticle (LNP)-encapsulated mRNA must be internalized by host cells and successfully released into the cytoplasm. Uptake is primarily mediated by endocytosis, particularly by antigen-presenting cells (APCs) such as dendritic cells, which play a pivotal role in initiating adaptive immune responses [97]. Following cellular internalization, the LNPs are sequestered within endosomes, where acidification triggers key biochemical transitions in their structure. At low pH, ionizable lipids within the LNP become protonated, facilitating endosomal escape through several proposed mechanisms: The proton sponge effect, in which an influx of protons, chloride ions, and water causes endosomal swelling and rupture and membrane destabilization and fusion, is enabled by conformational changes in the lipid structure under acidic conditions. Efficient endosomal escape remains a significant bottleneck in RNA delivery and is considered a major determinant of vaccine potency and intracellular translation efficiency [98,99,100].

#### 8.2.2. Intrinsic Adjuvant Properties of Lipid Nanoparticles

Beyond delivery, LNPs exhibit inherent adjuvant activity, primarily mediated by their ionizable lipid components. These lipids can activate innate immune pathways, leading to the production of inflammatory cytokines such as IL-6, IL-1β, and type I interferons (IFN-α) [33,101,102].

#### 8.2.3. Alternative Delivery Platforms

Although LNPs currently dominate the clinical landscape of RNA vaccines, their limitations have spurred the exploration of alternative delivery systems. These include polymer-based nanoparticles such as PLGA, cationic lipoplexes, and targeted nanocarriers designed to improve tissue specificity and reduce off-target effects [103]. Such platforms may address challenges associated with PEG-related hypersensitivity, off-target antigen expression, and stringent cold-chain requirements, broadening the applicability of RNA vaccines across different populations and geographies.

#### 8.2.4. Formulation Stability and Storage

A critical challenge in RNA vaccine formulation is the inherent instability of RNA molecules, which are susceptible to hydrolysis and degradation by ubiquitous RNases. To maintain efficacy, current mRNA vaccines often require freezing storage conditions, posing logistical obstacles for global distribution [20]. Efforts to improve thermostability include lyophilization techniques, the incorporation of stabilizing excipients, and the structural optimization of both lipids and mRNA [104,105]. These strategies aim to enable more flexible and accessible vaccine deployment, particularly in resource-limited settings.

#### 8.2.5. Biodistribution and Pharmacokinetics

Understanding the biodistribution and pharmacokinetics of lipid nanoparticles (LNPs) and the encoded antigens is critical for optimizing vaccine design, maximizing efficacy, and ensuring safety. Following intramuscular (IM) administration, LNPs remain localized at the injection site but gradually enter systemic circulation, reaching peak plasma levels around 4 h post-injection. Within 48 h, they accumulate primarily in well-perfused organs such as the liver, adrenal glands, spleen, and ovaries, with approximately 20% retention in the liver beyond this timeframe [106]. The route of delivery also influences localization; for example, intradermal injections tend to confine antigen expression more locally compared to IM or IV delivery [107].

A key driver of hepatic uptake is the adsorption of apolipoprotein E (ApoE) onto LNPs in the bloodstream, which mediates binding to LDL receptors on hepatocytes [108]. Consequently, LNP characteristics—specifically, size, surface charge, and lipid composition—directly influence their biodistribution and safety profile. For instance, smaller LNPs (~30 nm) can access a broader range of tissues but may exhibit inefficient endosomal escape and increased off-target toxicity, while medium-sized (~100 nm) particles strike a balance between retention at injection sites, lymphatic drainage, and systemic access. In contrast, larger particles (~300 nm) are cleared faster and show reduced cellular uptake [109]. These biodistribution dynamics underscore the critical need to minimize antigen expression in non-APC tissues (e.g., heart, brain, reproductive organs), which could otherwise provoke unintended immune responses [107].

#### 8.2.6. mRNA Optimization Strategies

In addition to delivery considerations, optimizing the mRNA construct itself is essential for precise control over immune activation, antigen production, and stability. Key strategies include the following:

- Nucleoside modifications, such as the incorporation of pseudouridine or N1-methylpseudouridine, which reduce innate immune sensing and enhance translational efficiency, improving both protein expression and vaccine tolerability [36,110,111,112]
- Codon optimization, aligning codon usage to human tRNA abundance to enhance translation without altering the amino acid sequence [113,114]
- Untranslated region (UTR) and poly(A) tail engineering, which increase mRNA stability and translation initiation.
- Advanced computational tools, such as LinearDesign, which simultaneously optimize secondary structure folding and codon usage, leading to increased mRNA half-life, protein expression, and antibody titers in animal models [95].

These multifaceted optimization approaches collectively aim to produce mRNA molecules that yield robust and sustained antigen expression while avoiding excessive innate immune activation and degradation. While RNA vaccines showcase high efficacy and a generally favorable safety profile, understanding the full spectrum of adverse events (AEs) and their immunological underpinnings is essential for continuous optimization and public health confidence.

### 8.3. RNA Vaccine Reactogenicity, Adverse Events and Safety Considerations

#### 8.3.1. Common Reactogenicity and Systemic Adverse Events

Vaccination, regardless of the platform, often triggers transient, self-resolving local and systemic reactions—collectively termed reactogenicity—that often serve as markers of innate immune activation. Clinical trials and post-marketing surveillance of mRNA vaccines (e.g., BNT162b2, mRNA-1273) consistently report local reactions like injection site pain, erythema, and swelling and systemic reactions like fatigue, myalgia, headache, chills, malaise, fever, and arthralgia [115,116]. The rate and severity of these adverse events tend to be higher following the second vaccine dose compared to the first, and higher vaccine doses have also been associated with increased reactogenicity [115]. Post-marketing surveillance has largely affirmed the robust safety profile observed in early trials, with reactogenicity profiles consistent with previous trials and comparable to other licensed vaccines.

The reactogenicity observed after mRNA vaccination is largely attributed to the inflammatory nature of the vaccine components, particularly the ionizable lipid component of the LNPs [117]. This lipid triggers the rapid release of high amounts of innate inflammatory cytokines (e.g., IL-1β, IL-6, GM-CSF, type I interferon), which are responsible for the acute systemic symptoms. Furthermore, manufacturing impurities, such as low levels of double-stranded RNAs (dsRNA) that can form during production, can also activate innate immune sensors and contribute to the inflammatory profile of the vaccine [118]. Variations in the amounts of mRNA-LNP or the mRNA:LNP ratio between different vaccine lots have been hypothesized as a potential explanation for observed differences in adverse event rates, underscoring the importance of strict purity criteria and consistent manufacturing processes [44].

#### 8.3.2. Immunological Mechanisms of Specific Adverse Events

While generally safe, rare but serious adverse events have been reported, and their immunological mechanisms are subjects of ongoing investigation.

#### 8.3.3. Myocarditis/Pericarditis

Myocarditis (inflammation of the heart muscle) and pericarditis (inflammation of the sac surrounding the heart) have been recognized as rare complications following COVID-19 mRNA vaccination, predominantly observed in young adult and adolescent males, typically occurring 2 to 3 days after the second dose. The precise immunological mechanisms are not fully elucidated, but several hypotheses have been proposed:

- **Molecular mimicry**: One leading hypothesis suggests molecular mimicry between the SARS-CoV-2 spike protein (encoded by the vaccine mRNA) and self-antigens present in cardiac tissue. Antibodies generated against the spike protein could potentially cross-react with structurally similar human peptide sequences, such as α-myosin, leading to an autoimmune attack on cardiac myocytes [119].
- **Aberrant Immune Response to mRNA/LNP:** In certain genetically predisposed individuals, the immune response to the vaccine’s mRNA or LNP components might be dysregulated. Although nucleoside modifications aim to reduce innate immunogenicity, some individuals may still mount an exaggerated inflammatory response, with dendritic cells or TLR-expressing cells releasing high levels of cytokines and activation markers. This could lead to a proinflammatory cascade that contributes to myocardial inflammation [120,121].
- **Off-target spike expression:** The biodistribution of LNPs is not strictly confined to immune cells or lymphoid organs; they can reach various tissues, including the heart [58]. If the vaccine mRNA is translated into spike protein in non-APCs within cardiac tissue, these spike-expressing cells could become targets for the antigen-specific adaptive immune response. This immune-mediated attack on healthy cells expressing the vaccine antigen could contribute to inflammation and damage in the affected organ [122].
- **Autoantibody production:** The generation of autoantibodies targeting cardiac antigens has also been proposed as a mechanism, although their direct pathogenic role versus being a consequence of inflammation is still debated [123].
- **NK cell dysregulation:** An increase in Natural Killer (NK) cell frequency has been observed in some cases, though these cells’ precise contribution to pathology or resolution remains unclear [124,125].

The occurrence of myocarditis is likely not due to a single mechanism but rather a convergence of factors, including the presence of the spike protein, the intrinsic immunogenicity of the mRNA/LNP, and individual genetic predispositions. The potential for off-target translation of the spike protein in non-APCs highlights the critical importance of controlling the biodistribution and cellular tropism of mRNA-LNPs to prevent antigen expression in vulnerable tissues. This understanding is essential for developing future vaccine designs that minimize such rare but serious adverse events.

#### 8.3.4. Anaphylaxis

Anaphylaxis is a rare but severe allergic reaction that can occur after vaccination, typically on first exposure [126]. The proposed immunological mechanisms for anaphylaxis associated with mRNA vaccines are complex:

- **Pre-existing Anti-Polyethylene Glycol (PEG) Antibodies:** Polyethylene glycol (PEG), a component of LNPs, is a primary suspect. Individuals may have pre-existing antibodies (IgM, IgG, or IgE) against PEG due to prior exposure to PEGylated products (e.g., cosmetics, medications). These antibodies can bind to PEG on the LNP surface, leading to complement activation (via IgM/IgG) or the crosslinking of Fc receptors on mast cells (via IgE/IgG), triggering rapid mast cell degranulation and the release of inflammatory mediators [127,128,129].
- **Direct Mast Cell Activation:** LNPs or their dispersed components may directly activate mast cells or basophils through various receptors, leading to degranulation independent of pre-existing antibodies [130].
- **Complement Activation-Related Pseudoallergy (CARPA):** The LNP itself can directly trigger complement activation, leading to the production of anaphylatoxins (C3a, C5a) that activate mast cells and basophils, resulting in pseudoallergic reactions [130,131,132].
- **Contact System Activation:** The negatively charged nucleic acid (mRNA) can potentially activate factor XII of the contact system, leading to bradykinin production, which can cause angioedema and anaphylactoid reactions.

Host factors also play a role, with anaphylactic reactions to mRNA vaccines being strikingly more common in females and individuals with a history of atopy or mast cell hyperresponsiveness [133,134].

#### 8.3.5. Neurological and Thrombotic Events

While most neurological symptoms following mRNA vaccination are mild and transient (e.g., headache), more severe neurological disorders have been reported, though their causal relationship with the vaccine is often complex to establish. These include Bell’s palsy, herpes zoster reactivation, acute transverse myelitis, acute disseminated encephalomyelitis, and Guillain–Barré syndrome. The underlying pathophysiology is not fully understood, but potential neuroimmune mechanisms are under investigation [135,136,137,138].

Rare but serious thrombotic events, such as thrombosis with thrombocytopenia syndrome, have been reported following vaccination, necessitating careful observation and management [139]. While more commonly associated with adenoviral vector vaccines, the potential for such events with mRNA vaccines requires ongoing pharmacovigilance [139,140].

### 8.4. Mitigation Strategies and Safety Considerations

Ensuring the highest level of safety for RNA vaccines involves continuous monitoring and strategic refinements in vaccine design and deployment. Continuous pharmacovigilance and robust real-world data analysis are essential for identifying and characterizing rare adverse events, understanding their incidence, and exploring potential causal relationships [141]. This ongoing monitoring allows for informed patient stratification, tailored safety assessments for specific subgroups (e.g., pediatric populations, older adults), and personalized vaccination recommendations [141,142]. Mitigation strategies for adverse events are multifaceted, focusing on refining vaccine components and manufacturing processes:

- **LNP Composition Refinement:** This includes exploring and implementing alternative LNP components that are less immunogenic or reactogenic. For instance, replacing PEG with zwitterionic materials like poly(carboxybetaine) (PCB) has shown promise in minimizing undesired immune activation while maintaining delivery efficiency [91,92,93].
- **Purity Criteria:** Ensuring strict purity criteria for the mRNA product is crucial to minimize contaminants such as double-stranded RNA (dsRNA) or DNA fragments, which can activate innate immune sensors and contribute to inflammation and adverse events [44].
- **Dose Optimization:** Adjusting vaccine doses, particularly for booster shots or in specific populations, can help balance immunogenicity with reactogenicity, as higher doses have been associated with increased adverse event rates [143].
- **Targeted Delivery:** Future LNP engineering efforts aim to achieve the more targeted delivery of mRNA to specific immune cells or lymphoid organs, thereby minimizing off-target antigen expression in sensitive tissues and reducing the potential for immune-mediated adverse events [20,144].

These strategies collectively aim to fine-tune the immune symphony orchestrated by RNA vaccines, maximizing protective efficacy while minimizing the risk of adverse reactions.

## 9. Current and Future RNA Vaccine Projects

The success of COVID-19 mRNA vaccines has catalyzed an explosion of research and development, extending the application of RNA vaccine technology far beyond infectious diseases into a broad spectrum of therapeutic areas.

### 9.1. Infectious Disease Vaccines

The rapid adaptability and potent immunogenicity of mRNA platforms make them ideal candidates for combating a wide range of infectious pathogens, including those with high variability or for which traditional vaccines have proven challenging.

Approved and next-generation COVID-19 mRNA vaccines (including circRNA and saRNA platforms): The BioNTech/Pfizer (BNT162b2) and Moderna (mRNA-1273) vaccines were the first nucleoside-modified mRNA vaccines to receive accelerated regulatory authorization, demonstrating remarkable efficacy (over 90% protection against symptomatic SARS-CoV-2 infection) and acceptable safety profiles [145,146]. Building on this success, next-generation RNA vaccine platforms are under development:

- **Self-amplifying RNA (saRNA) vaccines:** These vaccines encode not only the antigen but also viral replicase enzymes that enable the mRNA to replicate within the host cell, leading to higher and more prolonged antigen expression from a lower initial dose. This can potentially reduce vaccine dosing requirements and associated side effects. Several saRNA COVID-19 vaccine candidates are in clinical trials [147,148,149].
- **Circular RNA (circRNA) vaccines:** These are novel platforms that utilize circular RNA molecules, which are inherently more stable than linear mRNA due to their covalently closed loop structure, potentially leading to more sustained antigen expression and improved immunogenicity. One circRNA COVID-19 vaccine candidate is in preclinical development [150,151,152].

Clinical trials for influenza, RSV, HIV, VZV, HSV, Zika, and other viral/bacterial pathogens: The mRNA platform’s “plug and play” feature, allowing for swift design and adaptation to evolving pathogens, makes it highly attractive for various infectious diseases.

- **Influenza:** Multiple mRNA influenza vaccine candidates are in clinical trials, with some already in Phase 3 studies (e.g., Moderna’s mRNA-1010). These aim to improve vaccine effectiveness by increasing antigenic fidelity to circulating strains and enabling faster adaptation to seasonal changes compared to traditional egg-based manufacturing [153].
- **RSV (Respiratory Syncytial Virus):** mRNA RSV vaccines have shown robust immunogenicity and efficacy. Moderna’s mRNA-1345 RSV vaccine, for instance, has received expanded approval for younger adults with underlying health conditions, demonstrating comparable neutralizing antibody responses to those in older adults and durable efficacy against RSV-LRTD (lower respiratory tract disease). Preclinical studies have also shown strong humoral and cellular immunity without vaccine-enhanced respiratory disease (VERD) [154,155].
- **HIV (Human Immunodeficiency Virus):** Targeted vaccine strategies employing priming and heterologous boosting doses are showing promise in Phase 1 clinical trials. These strategies aim to guide the immune system through stages of antibody development to activate early immune responses relevant to broadly neutralizing antibodies (bnAbs), which can recognize and block a wide range of HIV variants [156].
- **VZV (Varicella Zoster Virus):** Unmodified mRNA VZV vaccines have demonstrated immunogenicity comparable to, or even superior to, the licensed Shingrix vaccine in preclinical models, inducing strong Th1-biased antibody and T cell responses [157].
- **HSV (Herpes Simplex Virus):** A trivalent mRNA vaccine targeting HSV-2 glycoproteins has shown protective efficacy in animal models and is considered a suitable candidate for human testing.
- **Zika Virus:** LNP-encapsulated modified mRNA vaccines for Zika virus have resulted in high seroconversion rates in Phase 1/2 human clinical studies [158,159].
- **Other Pathogens:** mRNA vaccines are also under development for a range of other viral pathogens, including Cytomegalovirus, Epstein–Barr virus, Chikungunya, Nipah, rabies, and metapneumovirus/parainfluenza virus. Attempts are also being made to develop mRNA vaccines against certain bacterial infections, such as Group A streptococcal antigen (GAS) and Group B streptococcus (GBS), though many are still in preclinical stages [160].

### 9.2. Cancer Vaccines

The application of mRNA technology in oncology represents one of the most exciting frontiers, offering the potential for highly specific and personalized cancer immunotherapies.

Personalized neoantigen vaccines and their immunological findings in clinical trials (e.g., melanoma, pancreatic, colorectal, breast, prostate, hepatocellular, esophageal cancers): mRNA cancer vaccines have emerged as a promising novel approach to cancer immunotherapy, offering high specificity, better efficacy, and potentially fewer side effects compared to traditional treatments. A key advantage is their ability to cover a variety of tumor antigens simultaneously and activate a broader range of T-cell responses through the co-delivery of human leukocyte antigen (HLA)-I and HLA-II molecules. Personalized neoantigen vaccines, which are tailored to the unique mutations (neoantigens) present in an individual patient’s tumor, are a major focus [161,162,163,164].

Numerous clinical trials are underway globally for mRNA cancer vaccines, primarily in early phases (Phase 1/2), targeting various cancer types:

- **Melanoma:** mRNA-4157/V940, a personalized neoantigen mRNA vaccine, in combination with pembrolizumab (an anti-PD1 immune checkpoint inhibitor) has advanced to Phase 3 clinical trials and shown a noticeably decreased risk of recurrence after surgery compared to pembrolizumab monotherapy [161,163].
- **Pancreatic Cancer:** Autogene cevumeran, another personalized mRNA neoantigen vaccine, when combined with Atezolizumab (anti-PD-L1) and chemotherapy, stimulated T-cell activity associated with delayed recurrence and significantly higher relapse-free survival in responders [162,164].
- **Brain Cancer (Glioblastoma):** A novel mRNA vaccine developed at the University of Florida, using a patient’s own tumor cells to create mRNA clusters, rapidly shifted “immune cold” tumors to “hot” (immunologically active) within 48 h in preclinical models and human patients, activating the early immune system against these aggressive cancers [165].
- **Hepatocellular Carcinoma (HCC):** A vaccine encoding nearly 20 frequently upregulated HCC antigens (ABOR2014) is in its first human clinical trial [163].
- **Other Cancers:** Clinical studies are also exploring mRNA vaccines for colorectal cancer, breast cancer, prostate cancer, esophageal cancer, leukemia, multiple myeloma, mesothelioma, renal cell carcinoma, ovarian cancer, and non-small cell lung cancer (NSCLC) [161].

mRNA cancer vaccines are not merely about delivering an antigen; they are powerful tools for actively reprogramming the tumor microenvironment and the host immune system. The ability to induce a rapid shift from an “immune cold” (immunosuppressive) to an “immune hot” (immunologically active) tumor environment and to enhance specific T cell subsets (e.g., through the co-delivery of cytokines like IL-12) represents a sophisticated manipulation of the immune symphony to overcome tumor-induced immunosuppression [161,163]. This suggests a future where mRNA vaccines are integral to combination immunotherapies, actively shaping the anti-tumor immune landscape for improved patient outcomes.

Combination strategies with other immunotherapies: A significant trend in cancer immunotherapy is the combination of mRNA vaccines with other therapeutic modalities, particularly immune checkpoint inhibitors (ICIs). ICIs, such as PD-1/PD-L1 blockers, work by unleashing existing anti-tumor T cell responses. mRNA vaccines can synergize with ICIs by expanding the anti-tumor T cell repertoire, promoting antigen release from tumor cells, and facilitating antigen presentation to the immune system [161]. This combinatorial approach has shown promising results in clinical trials, leading to enhanced anti-tumor effects and improved patient outcomes.

### 9.3. Other Therapeutic Applications

The versatility of mRNA technology extends beyond vaccines, holding significant promise for a wide array of other therapeutic applications. Emerging applications in protein replacement therapy, gene editing, and regenerative medicine include the following:

- **Protein Replacement Therapy:** For diseases caused by the absence or underexpression of specific proteins (e.g., genetic disorders, enzyme deficiencies), mRNA technology can deliver instructions for the in-situ production of the missing protein, offering a novel therapeutic approach [166].
- **Gene Editing:** mRNA can be used to deliver transiently expressed programmable nucleases (e.g., CRISPR-Cas9 systems) to correct harmful mutations or introduce protective genetic changes within cells, providing a powerful tool for gene editing without the risk of genomic integration associated with DNA-based methods [166].
- **Regenerative Medicine:** mRNA technology holds potential in regenerative medicine to replace, regenerate, or restore damaged tissues or cells. This could involve delivering mRNA encoding growth factors or other therapeutic proteins to promote tissue repair or cellular differentiation [166].

These emerging applications highlight the broad therapeutic potential of mRNA technology, positioning it as a versatile platform that could revolutionize the treatment of a wide range of diseases in the coming decades.

### 9.4. The Transformative Journey and Future Horizons of RNA Vaccine Technology

The journey of RNA vaccines from conceptualization to global deployment has been nothing short of revolutionary. The unparalleled speed with which mRNA vaccines were developed and authorized during the COVID-19 pandemic has demonstrated the immense potential of synthetic RNA platforms to reshape modern vaccinology [167]. This success stems from the ability of RNA vaccines to orchestrate a sophisticated immune symphony, leveraging both the mRNA payload and the lipid nanoparticle (LNP) delivery system to elicit potent and durable protective responses [168].

Significant advancements have been made in elucidating the intricate immunological mechanisms that underpin RNA vaccine efficacy. Pattern recognition receptors (PRRs), including endosomal Toll-like receptors (TLRs 3, 7, and 8) and cytoplasmic RIG-I-like receptors (RIG-I and MDA5), play a pivotal role in sensing exogenous RNA and initiating innate immune signaling cascades [169]. The strategic incorporation of nucleoside modifications, particularly pseudouridine, has proven essential in balancing enhanced mRNA stability and translational efficiency with a controlled dampening of innate immune sensing, thereby optimizing antigen expression without triggering excessive inflammation [169].

Beyond their role as delivery vehicles, LNPs have emerged as potent intrinsic adjuvants. The ionizable lipid components in LNPs drive the rapid induction of pro-inflammatory cytokines such as IL-1β, IL-6, IFN-α, and IFN-γ, creating an inflammatory milieu critical for activating professional antigen-presenting cells like dendritic cells [170]. This activation facilitates efficient antigen processing and presentation and promotes the differentiation of T follicular helper cells and the formation of germinal centers, processes indispensable for generating high-affinity antibody responses and long-lived memory B cells [170].

Importantly, RNA vaccines have been shown to induce not only classical adaptive memory but also a form of trained immunity in macrophages, characterized by persistent epigenetic and transcriptomic reprogramming that enhances responsiveness to subsequent challenges [48]. This phenomenon suggests broader, non-specific protective capacities that extend beyond antigen-specific responses. In parallel, detailed studies have mapped the differentiation of CD4+ T helper subsets and cytotoxic CD8+ T lymphocytes, as well as the establishment of central, effector, and tissue-resident memory T cell populations, underscoring the comprehensive nature of RNA vaccine-induced immunity [46,48].

Despite these achievements, substantial challenges remain. LNP optimization is needed to improve tissue targeting and minimize off-target effects that may contribute to rare but significant adverse events, including myocarditis, anaphylaxis, and neurological complications. Addressing these events requires a deeper understanding of host genetic predispositions, specific vaccine components such as polyethylene glycol (PEG), and the complex interplay of innate immune activation and dysregulation [118,171]. Furthermore, maintaining the long-term durability and breadth of protection remains an area of intense investigation, especially against rapidly evolving pathogens such as influenza and SARS-CoV-2 variants [172]. Recent advances in next-generation RNA platforms promise to overcome some of these limitations. Circular RNA vaccines are emerging as a novel approach, offering enhanced stability due to their resistance to exonuclease degradation and the potential for more sustained antigen expression [173,174,175]. Similarly, self-amplifying RNA vaccines, which encode their own replication machinery, have shown the capacity to achieve potent immune responses at lower doses compared to conventional mRNA, thereby expanding manufacturing efficiency and potentially reducing reactogenicity [176]. The clinical development of these platforms is progressing rapidly across multiple therapeutic areas [177].

Equally transformative are advances in delivery technologies. Novel approaches such as dendritic cell–targeting nanoparticles, tissue-specific delivery vehicles, and mucosal vaccine formulations for oral or intranasal administration hold the promise of improved efficacy, the induction of local immunity, and greater patient acceptance [178]. These strategies also align with the urgent need to expand vaccine accessibility, particularly in resource-limited settings where cold-chain storage remains a barrier [179]. The development of thermostable formulations and innovative distribution models will be essential for realizing the global health potential of RNA vaccines [180].

Beyond infectious diseases, the flexibility of the RNA platform heralds a new era of precision medicine. Personalized cancer immunotherapy is among the most promising frontiers, where RNA vaccines encoding patient-specific neoantigens can be combined with immune checkpoint inhibitors to reprogram immunologically “cold” tumors into responsive “hot” microenvironments [164]. Additionally, mRNA technology offers compelling possibilities for protein replacement therapy, the in-situ production of deficient enzymes, and the transient delivery of gene-editing tools such as CRISPR-Cas9 without the risks associated with permanent genomic integration. Research into the application of mRNA vaccines to induce immune tolerance in autoimmune diseases and to support regenerative medicine by encoding therapeutic growth factors continues to advance.

Regulatory frameworks have evolved rapidly to keep pace with these innovations. The experience of accelerated emergency authorizations during the pandemic has informed the development of more agile regulatory pathways that still maintain rigorous safety standards. However, manufacturing RNA vaccines requires specialized facilities, advanced analytical methods, and strict quality control protocols to ensure product consistency and potency [181]. The international harmonization of regulatory standards will be essential to enable timely global access and equitable distribution [181].

## 10. Conclusions

RNA vaccines have fundamentally transformed vaccinology by enabling unprecedented speed, scalability, and adaptability. The immune symphony they orchestrate—from innate sensing and inflammatory priming to adaptive memory generation—reflects decades of scientific innovation [20]. While challenges remain in improving delivery, enhancing stability, and ensuring equitable access, the development of next-generation RNA platforms and advanced formulation strategies promises to expand the impact of this technology across infectious disease prevention, cancer immunotherapy, genetic medicine, and beyond [182]. As research continues to unravel the complex immunological mechanisms that drive RNA vaccine efficacy, these insights will guide the creation of safer, more effective, and more accessible therapeutics, ultimately transforming the landscape of global health.

## Figures and Tables

**Table 1:** Overview of different optimization techniques for RNA vaccines.

Innovation	Effect on Dosing Frequency & Tailoring	Example	Reference
**RNA chemical modifications**	Extends RNA half-life and activity	N1-methylpseudouridine in mRNA	[5]
**Targeted delivery (LNPs, GalNAc, AOCs)**	Tissue-selective delivery and reduced frequency	Vutrisiran (GalNAc-siRNA)	[8]
**Self-amplifying/Circular RNA**	Longer-lasting protein expression	Preclinical saRNA and circRNA therapies	[9]
**Personalized RNA design**	Patient/disease-specific schedules and targeting	Nusinersen for spinal muscular atrophy	[10]
**Antisense oligonucleotides (ASOs) with improved chemistry**	Reduced immune responses, enhanced stability, and tailored dosing	Mipomersen for familial hypercholesterolemia	[10]
**Ligand conjugation for targeted delivery**	Specific organ targeting and lower and less frequent dosing	Lumasiran for acute hepatic porphyria	[11]
**Biodegradable polymer carriers**	Sustained release and reduction in dosing frequency	siRNA formulations for hypercholesterolemia	[12]
**Nanoparticle encapsulation**	Enhanced stability and bioavailability	Patisiran (Onpattro) for hATTR amyloidosis	[8]
**MicroRNA mimics and inhibitors**	Gene expression modulation with potential for personalized dosing	AMT-130 in Huntington’s disease (ongoing trial)	[13]
